# American Insight Into Strabismus Surgery Before 1838

**DOI:** 10.1177/1179172117729367

**Published:** 2017-09-11

**Authors:** Christopher T Leffler, Stephen G Schwartz, John Q Le

**Affiliations:** 1 Department of Ophthalmology, Virginia Commonwealth University, Richmond, VA, USA; 2 Department of Ophthalmology, Bascom Palmer Eye Institute, Miller School of Medicine, University of Miami, Naples, FL, USA

**Keywords:** Strabismus surgery, medical history, prosthetic eyes

## Abstract

English surgeon John Taylor attempted to perform strabismus surgery in the 18th century. The field languished until, in Germany, treatment of strabismus by cutting an extraocular muscle was proposed by Louis Stromeyer in 1838 and performed by Johann Friedrich Dieffenbach in 1839. According to traditional teaching, there has never been any proof that anyone in the United States thought of the idea of strabismus surgery before Stromeyer’s report. In 1841, American surgeon William Gibson wrote that he had cut extraocular muscles to treat strabismus several times beginning in 1818 but never published his cases. Gibson’s former trainee Alexander E Hosack of New York confirmed Gibson’s memory. Interestingly, Hosack’s family had a connection with the family of New York oculist John Scudder Jr (1807-1843), whose reported cure of strabismus by cutting some of the fibers of an extraocular muscle was described in newspapers throughout the United States in 1837. Thus, Scudder’s report preceded that of Stromeyer. Scudder’s claim cannot be verified, but his description could have influenced Stromeyer, and demonstrates that the idea of strabismus surgery did exist in America before 1838.

The era of strabismus surgery began with English surgeon John Taylor (1703-1772), who generally explained that he was cutting the nerves supplying the extraocular muscle.^1,2^ He may occasionally have succeeded in improving strabismus, perhaps by inadvertently cutting the muscle itself.^1,2^ However, he was accused of performing surgery on the deviating eye, patching the dominant eye, and declaring success when the deviating eye was used to fixate.^1,2^ This fraudulent incident is consistent with the controversy which generally surrounded Taylor.^3^

After Taylor, the field languished until Louis Stromeyer of Germany recommended cutting extraocular muscles to improve strabismus in 1838.^2^ Stromeyer presented the idea entirely as his own and is generally credited with first clearly proposing myotomy for strabismus.^2^ In October 1839, Stromeyer’s idea was performed on a living patient by Johann Friedrich Dieffenbach of Germany.^2^ The new operation swept through Europe immediately and reached North America in 1840.^4^

The purpose of our review was to determine whether anyone in the United States had thought of the idea of strabismus surgery before Stromeyer published the idea in 1838. As we review below, several authors claimed in the 1840s that the idea had already existed in the United States and therefore that Stromeyer did not have priority for its conception. However, these American authors never provided any proof. We supplemented an analysis of previous reviews of the history of strabismus surgery,^1–6^ with a search of a US newspaper database^7^ for terms such as “strabismus,” “squinting,” and “oculist.”

A former student of surgeon William Ingalls (1769-1851) of Providence wrote that Ingalls offered him strabismus surgery in 1812 or 1813, after carefully explaining the anatomy.^4,5^ The student’s father refused to consent to the surgery, and the account did not reveal whether Ingalls actually performed the surgery in any other patients.^4^ The former student’s account was published without any comment from the 72-year-old Ingalls.^5^

Surgeon William Gibson of Philadelphia wrote in 1841 that he had performed myotomy for strabismus 4 times, beginning in 1818 in Baltimore.^4^ Gibson reported that he thought of the idea himself. After Gibson moved to Philadelphia, one of his strabismus surgeries produced a large consecutive exotropia,^4^ and his chief, Philip Syng Physick, dissuaded Gibson from performing any additional strabismus surgeries.^6^

Gibson’s story was confirmed by his former student Alexander Eddy Hosack of New York.^6^ Hosack apparently had a long-standing interest in eye surgery. An 1834 proposal to establish an eye and ear infirmary in the eastern part of New York City listed Hosack as the surgeon.^8^ Moreover, in November 1840, Hosack witnessed John M Carnochan^9^ perform an early strabismus surgery in New York.

According to this conventional history, there is no proof of the idea of strabismus surgery in America before 1840. However, our search revealed that the idea of myotomy for strabismus was clearly articulated in America by an associate of the Hosack family in 1837, the year before Stromeyer’s book. The case report of New York oculist John Scudder Jr was reprinted in dozens of US newspapers and some medical journals.

John Scudder Jr (1807-1843) of New York was a museum proprietor, oculist, and ocularist. His father owned the American Museum, of which David Hosack, a physician and antiquarian and the father of Alexander, was a financial backer.^10^ The elder Hosack had published an essay on vision in which he noted that patients with “squint” typically have reduced vision in one eye and pondered the function of the extraocular muscles. The elder Scudder was an expert in taxidermy^11^ and presumably would have used artificial eyes for the animal specimens at the museum. When he died in 1821, his son was described as “about 14 . . . remarkably smart.”^12^ In 1823, Scudder^13^ published a 103-page guide to the museum. The 16-year-old already had an interest in medicine: an electrical machine was available in the museum for medical purposes, for an extra fee.^13^ Scudder attended the College of Physicians and Surgeons in New York from 1823 to 1825 but did not graduate.^14^ He studied under David Hosack, professor of Physic and Clinical Medicine.^15^

In his first advertisements, he declared himself an oculist. He once removed copper intraocular foreign bodies following discharge of a percussion cap.^16^

Scudder first became known for producing and setting prosthetic eyes. Although others in America had set artificial eyes,^17^ Scudder is the earliest identified oculist in America to produce them himself. He began this practice by 1823 or 1824,^18^ although the prostheses were not advertised until 1827.^19^ By 1829, Scudder had performed “the operation of setting” artificial eyes 560 times.^18^ The eyes were so realistic that they could “deceive the most skeptical.”^19^ Those who live at a distance could send “an exact painted likeness of the eye” and Scudder would return an enamel eye.^19^

Perhaps, his interest stemmed from his taxidermy work for animal exhibits in the museum. The eyes were well received in the medical journals.^20^ By optical illusions, the pupil appeared to dilate with oblique viewing or in dim light.^20^ One patient who received an artificial eye complained of eye symptoms to another doctor without disclosing that the eye was a prosthesis. The doctor could not tell that the eye was prosthetic and prescribed an ointment which damaged it. Thus, trained observers apparently had trouble telling that the eyes were prosthetic. Scudder wrote that the eyes could do everything the natural eye could do except to see.

In the placing of artificial eyes, Scudder first became interested in correspondence of the eyes. The artificial eye will “move with the real eye in any direction.”^19^ In addition, “It plays in its socket as naturally as the other, and corresponds with its slightest movements.”^21^ Ocular motility was attained by attaching the artificial eye to the diseased eye.^20^

Perhaps, it was a natural progression to try to address the misalignment of real eyes (strabismus). In 1832, in a notice titled “Squinting,” Scudder^22^ announced that he had “discovered a method . . . of curing the disagreeable imperfection of obliquity in vision.” Whether the cure was medical or surgical was not stated.

In 1833, Scudder^23^ claimed to have put his “remedy” for “squinting” into practice. Scudder showed to a newspaper editor an 11-year-old boy who had been “operated on by Dr. S. and restored to sight” after an injury in which “the bursting of a gun” had “inverted it in its socket. The eye was replaced and vision restored.”^23^ The most likely explanation is that Scudder simply placed an artificial eye.

Several events in early 1837 could have led to contact between Scudder and surgeon Alexander Hosack. First, Scudder operated on a young girl who had been sewing when the tip of a needle broke off and become embedded deep in her eye. Other physicians had given up their attempts to remove the needle tip when Dr Scudder provided “an anodyne” for pain relief and then “made a small incision with a delicate operating knife; then by means of a powerful magnet,” he extracted the needle fragment.^24^ Perhaps he used the magnet on display at the museum.^13^

Second, Scudder^25^ published a recently rediscovered 1816 letter to his father from David Hosack. The letter accompanied Hosack’s donation of the curtains from Mary, Queen of Scots’ bed to the museum.^25^ It is easy to imagine that if the younger Scudder and the younger Hosack did speak, the oculist with a longtime interest in curing squinting and the surgeon who had been taught how such an operation could work would turn to that subject.

In August 1837, Scudder practiced in Albany. He reported that while in nearby Ballston Spa, he had performed the surgical “cure of Squinting, or Strabismus” on “a young lady.”^26–28^ This was the first time he used the medical term strabismus. Scudder noted,

The operation was performed by cutting some of the fibres of the muscle which held the eye obliquely; the consequence was, that the opposite muscle immediately brought the eye in its proper line of vision, and the unpleasant deformity of squinting was instantaneously removed.^26–28^

Even if Scudder learned of strabismus surgery from Hosack, Scudder’s description contains a refinement not found in the writings of Gibson, Stromeyer, Dieffenbach, or early American writers: cutting only some of the fibers. Scudder described partial myotomy (or tenotomy), which today is hailed as a modern and “minimally invasive” approach to strabismus surgery.^29^ A complete transection of the muscle risks overcorrection, as Gibson and others noted. Scudder’s report was carried in dozens of American newspapers and several medical journals in America.^27–28^ However, “the whole faculty ridiculed” his idea.^30^

In a follow-up report, the editor in Ballston Spa indicated that no one in that town had firsthand knowledge of the surgery claimed by Scudder.^31^ Thus, no witnesses corroborated Scudder’s account, which was considered a hoax.

The idea for strabismus surgery was next published in America in April of 1840 when surgeon William Ludwig Detmold, who practiced in New York, attributed the idea to Stromeyer.^32^ Detmold did not credit Scudder, the fringe oculist and museum proprietor with priority for the idea.

One 1842 reviewer did credit Scudder with inspiring the Europeans, and claimed that an “ingenious oculist” had seen Scudder’s report in a foreign newspaper.^30^ This concept is not impossible because Scudder’s artificial eyes, magnet extraction, and other events were widely covered in the British press. Given the widespread publicity in America, it is possible the idea could have been carried to Europe by a doctor or even a member of the public.

In 1836, Scudder had been struck about the face and eye with a heavy cane in a fight in the barroom of Tammany Hall.^33^ He died in Albany in 1843, at the age of 36 years, a victim of addiction to rum. He was remembered as “. . . a man of good education, a wit, and, but for intemperance, would have been an honorable and useful member of society.”^34^

Similar to John Taylor, his predecessor in promoting strabismus surgery, Scudder not only was willing to use showmanship and possible outright deceit but also was a man of ideas who may have advanced the field. Scudder’s impressive museum catalog and manufacture of detailed artificial eyes demonstrate that he did have the creativity to contribute intellectually to the medical field. Some questions remain. Did Scudder conceive of myotomy for strabismus by himself, or did he simply refine an idea handed down indirectly from William Gibson? Was Stromeyer influenced by Scudder’s report? Perhaps we will never know. What Scudder’s report demonstrates unequivocally is that the idea of myotomy for strabismus was conceived of (and published) in the United States before Stromeyer published the idea in Germany in 1838.
